# *EGFR*基因突变状态未知NSCLC治疗——TKI还是化疗？

**DOI:** 10.3779/j.issn.1009-3419.2014.10.01

**Published:** 2014-10-20

**Authors:** 晓晴 刘, 晶 许

**Affiliations:** 100071 北京，解放军第307医院 Department of Lung Cancer, 307 Hospital of PLA, Beijing 100071, China

**Keywords:** 肺肿瘤, *EGFR*基因状态未知, EGFR-TKIs, 化疗, Lung neoplams, Unknown *EGFR* gene status, EGFR-TKIs, Chemotherapy

## Abstract

目前对于表皮生长因子受体（epidermal growth factor receptor, *EGFR*）基因状态明确的非小细胞肺癌患者，其治疗推荐已成共识：对*EGFR*基因突变患者，可优先选择表皮生长因子受体酪氨酸激酶抑制剂（epidermal growth factor receptor-tyrosine kinase inhibitors, EGFR-TKIs），其治疗可最大获益；*EGFR*基因野生型患者，无论一线二线均应首选化疗。但是在临床实践中，仍有大部分患者由于各种原因未行*EGFR*基因检测，对于这部分基因状态未知患者的治疗，本文以循证医学的视角，回顾了相关临床研究，建议*EGFR*基因状态未知人群的治疗应依据种族、病理特点精细化分、区别对待，以甄别EGFR-TKI治疗高获益人群，科学合理安排治疗顺序。

## Abstract (en)

At present, the treatment recommendations of advanced non-small cell lung cancer (NSCLC) with clear epidermal growth factor receptor (*EGFR*) gene status has reached a consensus: patients with *EGFR* mutation-positive NSCLC can give priority to epidermal growth factor receptor-tyrosine kinase inhibitors (EGFR-TKIs), which can maximize the benefit from the treatment; while the patients with wild-type *EGFR* gene should regard chemotherapy as first choice whether the first-line or second-line therapy. A large proportion of patients, however, have not received *EGFR* gene test for various reasons. In the paper, we will thoroughly review the related clinical trial from the point of view of evidence-based medicine, and suggest that the treatment of advanced NSCLC with unknown *EGFR* gene status should be distinguished according to their race and pathological characters in order to find out the patients who are most likely to benefit from EGFR-TKIs, and thus arrange the treatment sequence scientifically and rationally.

2004年，Paez及Lynch分别在*Science*和*N Engl J Med*上报告表皮生长因子受体（epidermal growth factor receptor, *EGFR*）基因突变可以预测EGFR酪氨酸激酶抑制剂治疗的敏感性^[1, 2]^。也许当时这个科学发现的重大意义及后续效应连作者本人都始料未及。回望十年，层见叠出的证据已然验证了这一毫无争议的结论，而最重要的是*EGFR*基因突变和表皮生长因子受体酪氨酸激酶抑制剂（EGFR tyrosine kinase inhibitors, EGFR-TKIs）疗效之间的关联开创了肺癌个体化治疗的先河，至今其贡献度和影响力在该领域依然“经典”，堪称“里程碑”。

目前非小细胞肺癌（non-small cell lung cancer, NSCLC）依据驱动基因*EGFR*突变状态分为*EGFR*突变型、*EGFR*野生型和*EGFR*状态未知型，在个体化治疗理念主导的当今，临床实践必须依据其*EGFR*基因状态选择治疗。

## 1 *EGFR*基因突变型患者治疗

对于晚期NSCLC患者*EGFR*突变人群，IPASS、First-SIGNAL、OPTIMAL、EURTAC、NEJ002、WJTO3405、LUX-lung3和LUX-lung6这八项临床研究的结果显示，*EGFR*突变人群一线采用EGFR-TKI治疗与化疗相比，能够提高患者的客观缓解率（objective response rate, ORR），延长患者的无进展生存期（progression free survival, PFS），改善患者生活质量（表 1）^[3-10]^。INFORM、SATURN维持治疗研究结果也显示，在4个-6个疗程的化疗后，疾病有效和稳定的患者，采用EGFR-TKI维持治疗，也可延长*EGFR*突变患者的PFS^[11, 12]^。鉴于此类证据，美国国立综合癌症网络（National Comprehensive Cancer Network, NCCN）指南已做明确推荐。

**1:** *EGFR*突变阳性NSCLC患者一线TKI对比化疗研究 Efficacy outcomes from clinical trials evaluating EGFR-TKI in the first-line treatment of NSCLCs

Study	Number	*EGFR* mutation type	ORR (%)	PFS (mo)	HR
IPASS	261	19Del/L858R+other (8%)	71.2 *vs* 47.3	9.8 *vs* 6.4	0.48
First-SIGNAL	42	19Del/L858R	84.6 *vs* 37.5	8.4 *vs* 6.7	0.61
WJTOG 3405	172	19Del/L858R	62.1 *vs* 32.2	9.6 *vs* 6.6	0.49
NEJGSG002	224	19Del/L858R+other (6%)	73.7 *vs* 30.7	10.8 *vs* 5.4	0.30
OPTIMAL	154	19Del/L858R	83.0 *vs* 36.0	13.1 *vs* 4.6	0.16
EURTAC	173	19Del/L858R	58.0 *vs* 15.0	9.7 *vs* 5.2	0.37
LUX-LUNG 3	308	19Del/L858R+other (11%)	61.0 *vs* 22.0	11.1 *vs* 6.9	0.58
LUX-LUNG 6	364	19Del/L858R+other	66.9 *vs* 23.0	11.0 *vs* 5.6	0.28
EGFR: epidermal growth factor receptor; EGFR-TKIs: epidermal growth factor receptor-tyrosine kinase inhibitors; NSCLC: non-small cell lung cancer; PFS: progression free survival; ORR: objective response rate; HR: hazard ratio.					

## 2 *EGFR*基因野生型患者治疗

IPASS研究对*EGFR*突变状态患者的分层分析结果和多项二线研究（表 2）中对于*EGFR*野生型患者的亚组分析结果一致认为，明确为*EGFR*野生型患者，无论一线或二线治疗均应首选化疗^[3, 13-17]^。

**2:** *EGFR*野生型NSCLC化疗比较TKI临床试验结果 Efficacy outcomes of clinical trials comparing EGFR-TKI with chemotherapy in NSCLC with wild-type *EGFR* gene

Study	Agent	Number	PFS (mo)	HR (95%CI)	OS (mo)	HR (95%CI)
INTEREST	Gefitinib Docetaxel	106 123	1.7 2.6	1.24 （0.94-1.64）	6.4 6.0	1.02 （0.78-1.33）
TITAN^*^	Erlotinib Docetaxel/Pemetrexed	75 74	1.4 2.0	1.25 (0.88-1.78)	6.4 4.5	0.85 （0.59-1.22）
TAILOR	Docetaxel Erlotinib	104 107	3.4 2.4	0.69 （0.52-0.93）	N	
DELTA	Erlotinib Docetaxel	109 90	1.3 2.9	1.45 （1.09-1.94）	9.0 10.1	0.98 （0.69-1.39）
CTONG0806	Pemetrexed Gefitinib	76 81	4.8 1.6	0.51 （0.36-0.73）	N	
IPASS *EGFR* wild-type	Gefitinib Paclitaxel/Carboplatin	91 85	1.5 5.5	0.85 （2.05-3.98）	11.2 12.7	1.18 （0.86-1.63）
OS: overall survival; N: not acquired.						

总之，依据当前循证医学证据，*EGFR*基因突变状态明确的NSCLC患者，无论是突变还是野生型，治疗时机是一线抑或二线，其治疗路径基本清晰，治疗选择明朗，似乎已无悬念，且已达成共识并形成指南和规范，指导临床实践。

## 3 *EGFR*基因突变状态未知患者治疗：化疗还是EGFR-TKI？

“理想很丰满，现实很骨感”，现代人经常调侃的一句话，倒是形象、真实反映了目前我国肺癌临床实践中*EGFR*基因状态检测面临的诸多问题。中山肿瘤医院张力对全国NSCLC治疗状况的调查中发现，对于初诊的NSCLC患者，2010年*EGFR*突变检测率小于10%，到2012年*EGFR*突变检测率也仅提高到大约20%，这就意味着在中国约80%的NSCLC患者*EGFR*基因突变状态未知^[18]^。这使临床工作陷入徘徊、犹豫、不知所措的境地。那么对那些主客观条件无法检测*EGFR*基因突变状态的NSCLC患者，如何取舍和选择治疗：化疗还是EGFR-TKI？

不妨我们先回顾下*EGFR*基因突变状态未知的相关临床研究：

### 3.1 一线研究

TORCH是一项在西方国家进行的也曾是2010年美国临床肿瘤学会（American Society of Clinical Oncology, ASCO）年会令人瞩目的研究，该研究为非选择人群：一组进行厄洛替尼150 mg/d，直至疾病进展换健择/顺铂化疗6周期（E-GP），一组进行标准的健择/顺铂化疗6周期，直至疾病进展后换厄洛替尼治疗，主要终点为总生存期（overall survival, OS），次要终点为毒副作用、缓解率和PFS。在总体人群中，E-GP组中位OS为8.7个月，而GP-E组中位OS为11.6个月，具有统计学差异（*P*=0.000, 04）。该研究说明靶向治疗的研究应当考虑入组人群的*EGFR*突变构成比。由于TORCH研究主要纳入了*EGFR*突变比率低的西方人群作为非选择人群，可能对结果有影响^[19]^。

IPASS是在亚洲进行的一项多中心、随机Ⅲ期临床研究，总共随机入组了1, 217例患者，均为亚裔、不吸烟或轻度吸烟的Ⅲb期/Ⅳ期初治的腺癌患者。该项研究比较了一线应用吉非替尼和紫杉醇/卡铂的临床疗效，主要观察指标为PFS。总体人群中，吉非替尼组和紫杉醇/卡铂组的中位PFS分别为5.7个月和5.8个月，1年无进展生存率吉非替尼组为24.9%，化疗组为6.7%（HR=0.74, 95%CI: 0.65-0.85, *P* < 0.001）；吉非替尼组和紫杉醇/卡铂组的中位OS分别为18.8个月和17.4个月（HR=0.90, 95%CI: 0.79-1.02, *P*=0.109）。ORR吉非替尼组为43.0%，化疗组为32.2%（*P* < 0.001）。结果说明，在*EGFR*基因状态未知人群中（但主要是*EGFR*基因突变优势人群），一线选择吉非替尼比化疗复发风险降低，死亡风险更低，且有更好的ORR。当然由于该研究入组的主要为*EGFR*基因突变优势人群，故其结果并不能针对*EGFR*基因状态未知的所有人^[3]^。

FAST-ACTII设计初衷是了解交替治疗是否可以用于*EGFR*突变未知人群？其研究设计是既往未经治疗的Ⅲb期/Ⅳ期NSCLC，随机分到试验组吉西他滨（d1、d8）+顺铂/卡铂（d1）+厄洛替尼（d15-d28），q4w，共6个周期，之后厄洛替尼150 mg/d维持治疗至疾病进展；对照组是吉西他滨（d1、d8）+顺铂/卡铂（d1）+安慰剂（d15-d28），q4w，共6个周期，之后安慰剂维持至疾病进展。主要研究终点为PFS。研究结果显示，化疗联合EGFR-TKI组的PFS长于化疗联合安慰剂组（HR=0.57, 95%CI: 0.47-0.69; *P* < 0.000, 1）；OS也延长（HR=0.79, 95%CI: 0.64-0.99; *P*=0.042, 0）。在入组研究的451例患者中，有210例是*EGFR*突变状态未知的患者，其中104例入化疗联合EGFR-TKI组，106例为化疗联合安慰剂组。*EGFR*突变状态未知患者的亚组分析显示，化疗联合EGFR-TKI组*vs*化疗联合安慰剂组的PFS为7.1个月*vs* 6.0个月（HR=0.61, *P*=0.001, 1）；OS为18.1个月*vs* 16.2个月（HR=0.98, *P*=0.643, 1）。化疗联合EGFR-TKI组的毒副反应的发生率、严重程度均与化疗联合安慰剂组相当，而化疗联合EGFR-TKI组中患者的生活质量更好。从研究结果来看，交替模式具有很好的耐受性，且全组人群和EGFR状态未知人群PFS均能受益，此模式适用于EGFR状态未知的患者^[20]^。

对于*EGFR*基因状态未知的一线NSCLC患者，这三项在不同地区非选择人群中进行的化疗和TKI治疗对决，似乎结论并非一致，究其原因应是在选择富含*EGFR*基因突变人群比例方面的差异所致。

### 3.2 二线研究

INTEREST研究是一项比较易瑞沙与多西他赛治疗复治晚期NSCLC患者的全球多中心、前瞻性、随机对照Ⅲ期临床研究，全组共1, 466例患者，研究随机分为易瑞沙250 mg/d组和多西他赛75 mg/m^2^组，主要终点是总生存期。研究显示对于总体人群而言，二线吉非替尼和多西紫杉醇的疗效类似，1年生存率分别为32%和34%，中位生存时间（median survival time, MST）分别为7.6个月和8.0个月。然而进一步分析*EGFR*突变状态与吉非替尼和多西紫杉醇疗效的关系则发现：对于*EGFR*突变者，吉非替尼的有效率明显高于多西紫杉醇，分别为42.1%和21.1%（*P*=0.04）。PFS也明显长于多西紫杉醇，HR为0.16（*P*=0.001）；对于*EGFR*野生型患者，吉非替尼和多西紫杉醇无论疗效还是PFS均无明显差别^[13]^。

DELET（*J Clin Oncol* 2014年5月14日在线）是一项开放性的试验，301例Ⅲb期或Ⅳ期的NSCLC患者，既往经过1次或2次化疗，有可评估或可测量的病灶，体能状态（performance status, PS）评分在1分-2分之间，随机分配到厄洛替尼和多西他赛组。未检测*EGFR*状态人群结果：中位PFS厄洛替尼组为2.0个月，多西他赛组为3.2个月（HR=1.22, *P*=0.09）。中位随访8.9个月以后，疾病进展或死亡在厄洛替尼组和多西他赛组的发生率分别为94%和91.4%。中位OS为14.8个月*vs* 12.2个月（HR=0.91, *P*=0.53），有效率为17% *vs* 17.9%（*P*=0.88）。亚组分析PFS显示在两组之间无明显差异。除了组织学为腺癌的患者（HR=1.6, *P*=0.03）所有危险均有利于多西他赛组。依据*EGFR*突变状况分层的结果显示：在*EGFR*野生型的肿瘤患者中，厄洛替尼组的PFS是1.3个月，多西他赛组的PFS是2.9个月（HR=1.57, *P* < 0.01）。中位OS在厄洛替尼组是9.0个月，在多西他赛组是10.1个月（*P*=0.91）。有效率在厄洛替尼组是5.6%，在多西他赛组是20.0%（*P* < 0.01）。*EGFR*突变患者中，厄洛替尼组PFS为9.3个月，多西他赛组为7.0个月（HR=0.82, 95%CI: 0.43-1.53）。厄洛替尼组OS没有得到，多西他赛组的OS为27.8个月（HR=0.38, 95%CI: 0.10-1.10）。研究者得出结论：本研究显示在未行*EGFR*基因检测的NSCLC患者中，对比多西他赛和厄洛替尼，在PFS和OS上无明显差异^[16]^。

其他几项*EGFR*基因突变状态未知NSCLC中进行的TKI对照化疗研究显示，EGFR-TKI与化疗疗效相似，但安全性更好。而对于亚裔、腺癌患者，TKIs比化疗获益更明显，为二线治疗更优的选择（表 3）^[13, 14, 16, 21-23]^。

**3:** *EGFR*基因状态未知的NSCLC化疗对比TKI的临床试验结果 Efficacy outcomes of clinical trials comparing EGFR-TKI with chemotherapy in NSCLC with unknown *EGFR* gene statuss

Study	Agent	Number	PFS (mo)	HR (95%CI)	OS (mo)	HR (95%CI)
INTEREST	Gefitinib Docetaxel	733 733	2.2 2.7	1.04 (0.93-1.18)	7.6 8.0	1.02 (0.91-1.15)
V15-32	Gefitinib Docetaxel	245 244	2.0 2.0	0.90 (0.72-1.12)	11.5 14.0	1.12 (0.89-1.40)
ISTANA	Gefitinib Docetaxel	82 79	3.3 2.4	0.73 (0.53-1.00)	14.1 12.2	0.87 (0.61-1.21)
HORG	Erlotinib Pemetrexed	166 166	3.6 2.7	N	7.9 8.9	N
TITAN	Erlotinib Docetaxel/Pemetrexed	203 221	1.4 2.0	1.19 (0.97-1.46)	5.3 5.5	0.96 (0.78-1.15)
DELTA	Erlotinib Docetaxel	151 150	2.0 3.2	1.22 (0.97-1.55)	14.8 12.2	0.91 (0.68-1.22)

## 4 *EGFR*基因突变状态未知患者需精细划分、区别对待

在分子生物学指标指导下的个体化治疗时代，我们依然不能抛开患者的临床病理特点而不顾，因为正是具备某些特点的患者实质上蕴含了独特的分子事件。

PIONEER是一项在亚洲新诊断晚期肺腺癌患者中评估*EGFR*突变发生率以及对*EGFR*突变发生率有影响的人口学和临床因素的流行病学调查研究，是迄今最大的亚洲*EGFR*突变流行病学研究，共入组1, 482例患者，结果显示：亚裔肺腺癌患者*EGFR*基因突变率可高达55%。其中中国大陆入组的747例患者（741例可评估）其*EGFR*突变率为50.2%，这就提示中国每2例晚期肺腺癌患者中就有1例携带*EGFR*基因敏感突变。如果是不吸烟、腺癌患者，*EGFR*突变率达到60.7%，提示临床上存在大量潜在*EGFR*突变的复治腺癌患者，而他们正是TKIs治疗的获益人群^[24]^。INTEREST研究和KCSG-LU08-01研究即是最好的佐证^[13, 25]^。

INTEREST研究是一项比较易瑞沙与多西他赛治疗复治晚期NSCLC患者的全球多中心、前瞻性、随机对照Ⅲ期临床研究，全组共1, 466例患者，其中亚裔亚组323例，中国亚组222例。研究随机分为易瑞沙250 mg/d组和多西他赛75 mg/m^2^组，主要终点是OS。研究显示对于总体人群而言，二线吉非替尼和多西紫杉醇的疗效类似。在中国亚组中，腺癌患者共145例，在这些患者中，易瑞沙组23.29%的患者出现了客观缓解，高于多西他赛组的8.33%，提高幅度达到180%（*P* < 0.000, 1）。易瑞沙组的中位PFS达到5.4个月，长于多西他赛的3.9个月，绝对值延长1.5个月，提高幅度达到38%（*P* < 0.000, 1）^[13]^。

KCSG-LU08-01是一项比较易瑞沙与培美曲塞二线治疗腺癌不吸烟的韩国晚期NSCLC患者的随机Ⅲ期临床研究。研究总共入组133例患者，年龄≥18岁，东部肿瘤协作组（Eastern Cooperative Oncology Group, ECOG）PS 0分-2分，所有患者均为腺癌，不吸烟，既往接受过一种含铂方案治疗，并具有可测量病灶，随机分为易瑞沙组与培美曲塞组，主要研究终点为PFS。结果显示，易瑞沙组显著提高ORR达到58.8%，是培美曲塞组（22.4%）的近3倍（*P* < 0.001）。易瑞沙组中位PFS 9.4个月，明显长于培美曲塞组的2.9个月（*P*=0.006）^[25]^。

上述两项研究说明，突变未知的亚裔腺癌患者，二线治疗选择EGFR-TKI较化疗在疗效、安全性、生活质量上均有获益。所以对于*EGFR*基因状态未知人群，要依据种族、临床病理特点进行精细划分，以筛选出TKIs治疗获益者，千万不要让这些潜在的突变患者丧失使用EGFR-TKI的机会。

## 5 小结

分子生物学指标指导下的精准治疗大势所趋，未来首先要提高医患送检意识，尽可能创造条件，明确患者的*EGFR*基因状态，依据突变结果给予其最恰当、最有效的治疗，实现对NSCLC患者的个体化治疗。

对于*EGFR*基因状态未知人群，不能等量齐观、一概而论，建议对种族、临床病理特点进行精细划分，以甄别高突变人群或TKIs治疗获益人群；依据现有临床研究结果，科学合理安排化疗和TKIs治疗顺序，使之最大化从TKI治疗中获益。
